# Truncating *ARL6IP1* variant as the genetic cause of fatal complicated hereditary spastic paraplegia

**DOI:** 10.1186/s12881-019-0851-6

**Published:** 2019-07-04

**Authors:** Salma M. Wakil, Safa Alhissi, Haya Al Dossari, Ayesha Alqahtani, Sherin Shibin, Brahim T. Melaiki, Josef Finsterer, Amal Al-Hashem, Saeed Bohlega, Anas M. Alazami

**Affiliations:** 1 Department of Genetics, Research Centre, King Faisal Specialist Hospital & Research Centre, MBC-03, P.O. Box 3354, Riyadh, 11211 Saudi Arabia; 2 Saudi Human Genome Program, King Abdulaziz City for Science and Technology, Riyadh, Saudi Arabia; 3 Department of Neurology, Krankenanstalt Rudolfstiftung, Messerli Institute, Postfach 20, 1180 Vienna, Austria; 4 Prince Sultan Riyadh Military Medical City, Riyadh, Saudi Arabia; 5 Department of Neurosciences, King Faisal Specialist Hospital & Research Center, Riyadh, Saudi Arabia

**Keywords:** Hereditary spastic paraplegia, Autosomal recessive, Whole exome sequencing, ADP ribosylation factor like GTPase 6 interacting protein 1

## Abstract

**Background:**

Mutations in *ARL6IP1*, which encodes a tetraspan membrane protein localized to the endoplasmic reticulum (ER), have been recently described in a large family with a complicated form of hereditary spastic paraplegia (HSP).

**Case presentation:**

We sought to expand the HSP phenotype associated with *ARL6IP1* variants by examining a Saudi kindred with a clinically more severe presentation, which resulted in spontaneous neonatal death of both affected siblings. Clinical features encompassed not only spastic paraplegia but also developmental delay, microcephaly, cerebral atrophy, periventricular leukoencephalopathy, hypotonia, seizures, spasticity, jejunal stricture, gastrointestinal reflux, neuropathy, dysmorphism and respiratory distress. We performed clinical assessment and radiological studies of this family, in addition to homozygosity mapping and whole exome sequencing (WES) to identify the disease-associated variant. Homozygosity mapping localized the causative gene to a region on chromosome 16 harboring *ARL6IP1*. WES of the index case identified the homoallelic nonsense variant, c.112C > T in *ARL6IP1* that segregated with the phenotype and was predicted to result in loss of the protein. Allelic expression analysis of the parents demonstrated downward pressure on the mutant allele, suggestive of nonsense-mediated decay.

**Conclusions:**

Our report shows that the phenotype associated with *ARL6IP1* variants may be broader and more acute than so far reported and identifies fatal HSP as the severe end of the phenotypic spectrum of *ARL6IP1* variants.

## Background

Hereditary spastic paraplegia (HSP) represents a highly heterogeneous group of complex neurological disorders involving multiple loci and causative genes. HSP is clinically characterized by progressive weakness and spasticity, predominantly in the lower limbs, and is further subdivided into a pure and a complex form [1]. OMIM has assigned identifiers to 79 genes or loci that have been linked to spastic paraplegia (SPG1-SPG79, in order of their discovery), although this list is not comprehensive and continues to expand [2–4]. All modes of inheritance have been reported.

The human ADP ribosylation factor like GTPase 6 interacting protein 1(*ARL6IP1*) gene, situated at locus 16p12.3 encodes a tetraspan membrane protein that regulates intracellular trafficking pathways in the endoplasmic reticulum (ER) membrane [5]. ARL6IP1 acts as an anti-apoptotic protein specific to multicellular organisms, and is a potential player in shaping the ER tubules in mammalian cells. In neurons it has been associated with regulation of glutamate, a major excitatory neurotransmitter in excitatory synapse [6]. In drosophila, knockdown of the gene leads to progressive motor deficit [7].

Recently an *ARL6IP1* variant has been reported in a patient with spastic paraplegia, motor and sensory polyneuropathy and acromutilation [8]. Here, we report a lethal homoallelic truncating variant in *ARL6IP1* manifesting with classical features of HSP and additionally with dysmorphic features, developmental delay, microcephaly, neuropathy, leukoencephalopathy, partial agenesis of corpus callosum, seizures, high grade gastroesophageal reflux disease, and respiratory distress in two affected members of a consanguineous Saudi family. This presentation of features expands the phenotypic spectrum of *ARL6IP1*-associated HSP.

CARE guidelines were followed in this report.

### Case presentation

**Fig. 1:**
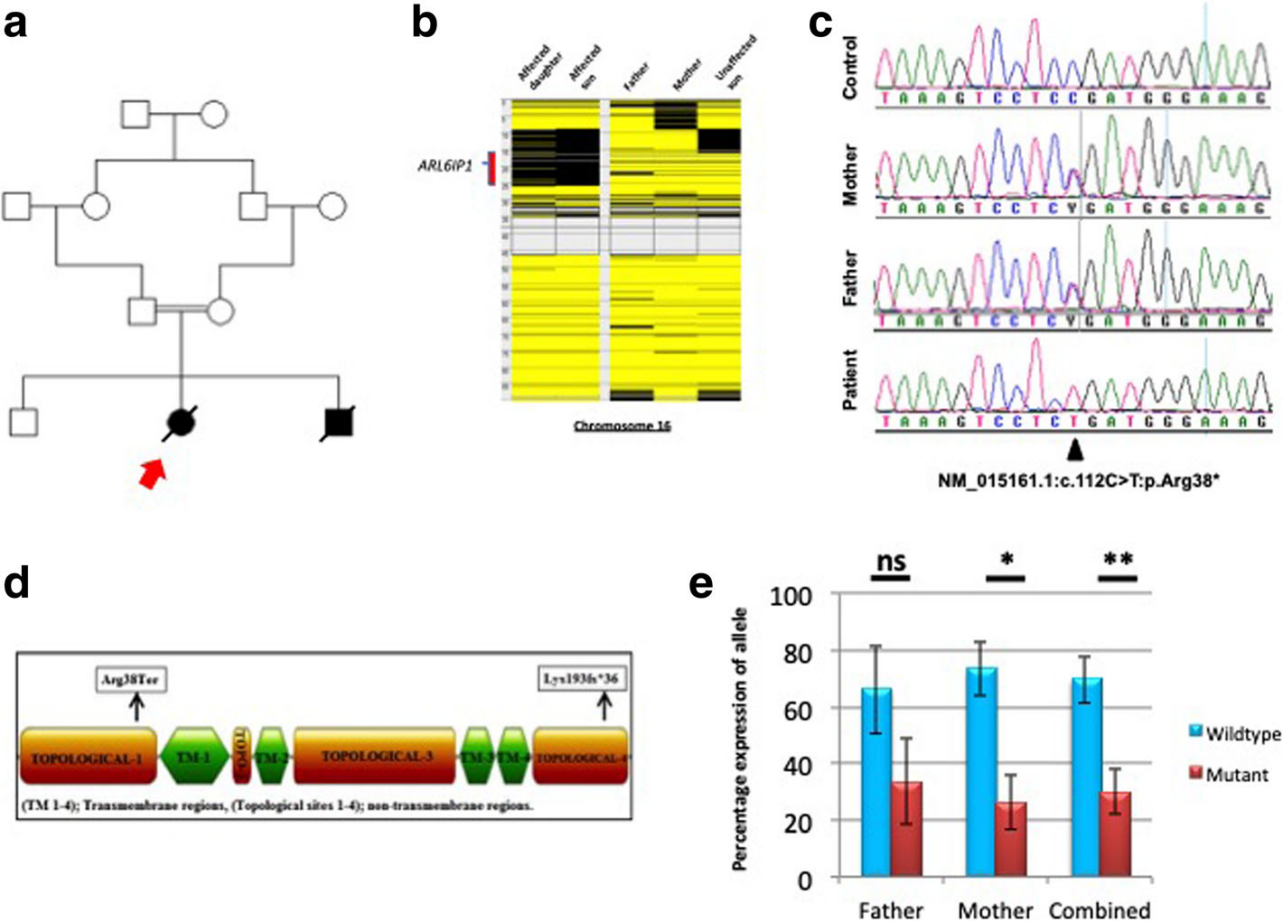
**a**. Pedigree of the family from this study. The index case (whose DNA was subjected to WES) is indicated with an arrow. **b** AutoSNPa software showing block of homozygosity (black) of family on chromosome 16 encompassing the *ARL6IP1* gene. Yellow regions indicate heterozygous SNPs. **c** Sequence electropherogram traces showing the mutation c.112C > T in one patient compared with both parents and a normal control. **d** Gene domains of *ARL6IP1* showing mutations reported thus far. **e** Analysis of the wildtype vs mutant allelic expression for both parents, based on three independent low-cycle RT-PCR reactions cloned into TOPO vector. Asterisks indicate significance levels (*p < 0.05, **p < 0.01, ns = not significant)

**Fig. 2:**
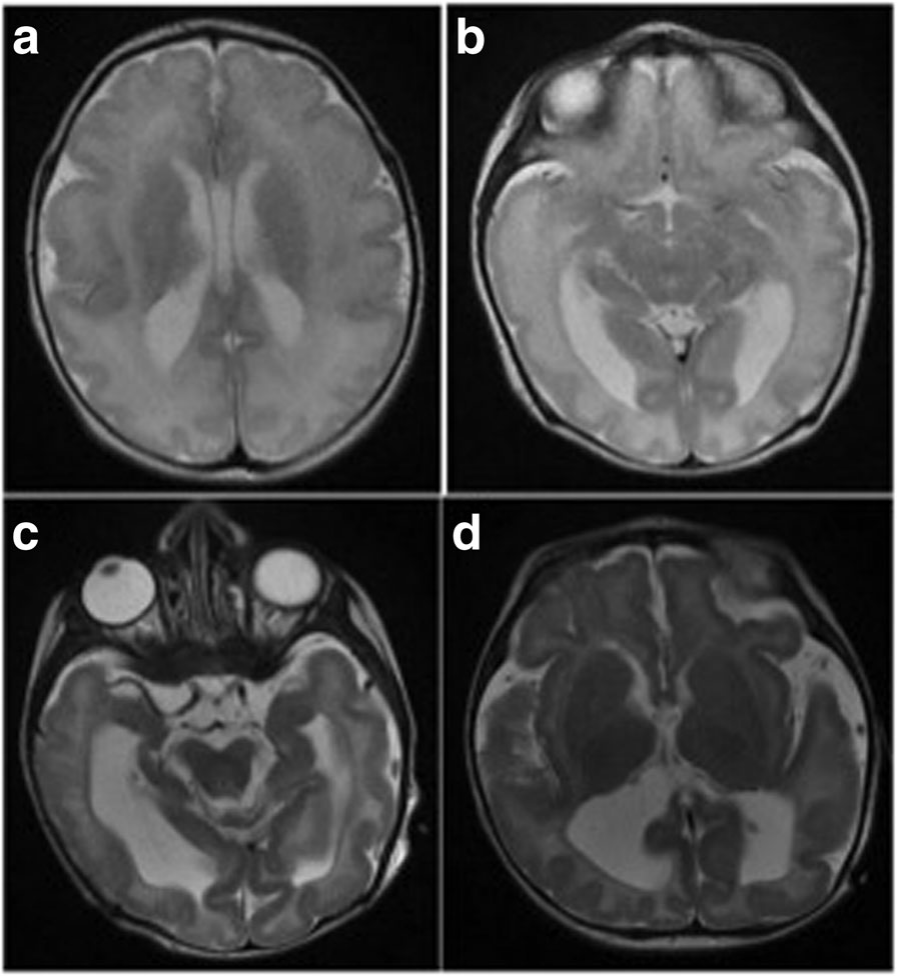
T2-weighted axial images of the cerebrum showing diffuse hyperintensity and atrophy of white matter (**a**, **b**, **c**, **d**), dilated ventricles (**b**, **c**, **d**), enlarged subarachnoid space (**c**, **d**), and partial agenesis of the corpus callosum (absence of splenium) (**d**)

Patient 1 was a 27 month old girl (Fig. 1a) born at full term pregnancy but too small for gestational age: her birth weight was 2.12 kg (< 10th centile), length 48 cm, and head circumference 31 cm (< 10th centile). She presented with failure to thrive, dysmorphic features including microcephaly (z score: − 2.4), severe lumbar hyperlordosis, limited hip abduction and neuropathy. Shortly after birth the patient developed respiratory distress requiring intubation. Several attempts of extubation were frustrating and the patient underwent tracheostomy. She had episodes of seizures controlled by phenobarbital. Since EEG did not show epileptiform discharges, phenobarbital was discounted without recurrence of seizures. Cerebral MRI revealed atrophy with dilated ventricles and enlarged subarachnoid space, extensive periventricular leukoencephalopathy, and partial agenesis of the corpus callosum (Fig. 2). Echocardiography revealed a patent foramen ovale and a patent ductus arteriosus. At age 1 month she developed gastrointestinal reflux due to intestinal obstruction secondary to jejunal stricture, managed by resection of the stricture and end-to-end anastomosis. Gastroscopy revealed a high-grade reflux disease. The index patient continued to have severe delay in all developmental aspects, did not gain any milestones, and continued to be ventilation-dependent. The further course was complicated by chronic lung disease. At 24 months she experienced an episode of apnea, and died from cardiac arrest at age 28 months. The karyotype was XX.

Patient 2, the brother of patient 1 (Fig. 1a), was delivered by elective Caesarian section at gestational week 37 because of placenta previa. He was admitted to the NICU because of hypotonia and respiratory distress. On examination, his birth weight was 2.3 kg (between the 10th and 50th percentile). Length was 48 cm (at the 50th percentile), and he had microcephaly (head circumference 32 cm at 2.6 centile, Z score − 1.9). He had overriding sutures, small fontanels, prominent nasal bridge, thin lips, microcephaly plagiocephaly, retrognathia, high arched palate, long hands, long fingers, hyper-extendable joints, redundant skin, large feet, severe head lag, absent deep tendon reflexes, and generalized hypotonia. Later in the course, he developed spasticity. Cerebral 1.5 T MRI was normal as well as metabolic screen (tandem MS, urine GCSM, ammonia, lactate, pyruvate, CK). Upon nerve conduction studies supramaximal stimulation did not evoke a motor response in any of the investigated nerves. Needle EMG revealed a neurogenic pattern. He required naso-gastric tube feeding and did not gain weight despite high caloric feeding. He required nasal cannula flow, HB-CPAP, and at age 52 days he was intubated. Extubation was frustrating several times. At times with progressive increase of oxygen demand he required high ventilator support. The further course was complicated by the development of pulmonary compromise, such as chronic lung disease and atelectasis on thoracic CT and ventilator-associated pneumonia. A study of the upper gastrointestinal tract showed gastro-esophageal reflux disease (GERD) managed with anti-reflux formula and medications. The patient eventually died at age of 16 months. The karyotype was XY.

The parents were first-degree cousins, and the family history was positive for neonatal deaths of individuals with joint contractures who died from respiratory distress.

Initial genetic workup for both patients involved molecular karyotyping using Cytoscan HD (Affymetrix, Santa Clara, USA), which contains 2.6 million markers of which 750,000 are genotyping single nucleotide polymorphisms and the remainder are non-polymorphic probes for genome coverage. Data analysis, using Chromosome Analysis Suite version Cyto 2.0.0.195(r5758), indicated that all individuals’ karyotypes were normal.

We next carried out homozygosity analysis via the Affymetrix Axiom array (Affymetrix, Santa Clara, CA, USA). All sets of homozygous stretches of SNPs were analyzed for shared runs of homozygosity (ROH) using autoSNPa (http://dna.leeds.ac.uk/autosnpa/), with a pre-set minimum 2 Mb size limit. Utilizing the five available family members, homozygosity data revealed a single ROH on chromosome 16 that was exclusive to the two affected members of this family (Fig. 1b). These findings were further interrogated using WES. DNA from the index case was treated to obtain an Ion Proton AmpliSeq library, which then underwent emulsion PCR on an Ion OneTouch System. Templated Ion Sphere particles were enriched using Ion OneTouch ES (Life Technologies, Carlsbad, CA, USA). The template-positive Ion PI Ion Sphere particles were processed for sequencing on the Ion Proton instrument (Life Technologies, Carlsbad, CA, USA); reads were mapped to UCSC hg19 (http://genome.ucsc.edu/) and variants identified using the Ion Torrent pipeline (Life Technologies, Carlsbad, CA, USA).

WES resulted in (on average) > 100x coverage totaling 46.2 Mb of genomic sequence from the index case. The resultant variant caller file (VCF) was filtered as follows. Given that the parents were consanguineous and asymptomatic, and given the presence of strong family history and the similarity of clinical presentation between both affected, only homozygous variants were considered in our analyses. Additionally, variants were excluded if they exhibited a high minor allele frequency (MAF > 0.01), were previously reported variants (present in dbSNP, 1000 genomes, 1500 Saudi exomes) or were present in 1200 chromosomes from the Saudi population. Only one homozygous variant survived the filtration process: *ARL6IP1* (NM_015161.1: c.112C > T: p.Arg38*) (Fig. 1c). Sanger sequencing on an ABI 3730xl automated sequencer (Applied Biosystems, Foster City, CA) confirmed that the variant segregated with the disease state.

This variant causes an arginine residue to be changed into a stop codon at position 38 (p.Arg38*) in the topological domain of ARL6IP1 (Fig. 1d). Given the truncating nature of the variant we wanted to assess whether the affected alleles were undergoing nonsense-mediated decay (NMD). Since no patient RNA was available, we examined the expression ratios of the wild type versus mutant alleles in the two obligate carriers (the parents). Total RNA was extracted from Paxgene tubes (PreAnalytix, Switzerland) then converted to cDNA. Reverse transcriptase (RT) PCR primers (5′-AAGAACAGCTGCAAGGATGG-3′ and 5′-GGTTGTGGACTTGTTGTCCC-3′) were used to amplify a region incorporating the mutation site. The amplification was conducted for a low number of cycles (18–20) to ensure that the reaction did not reach saturation levels. RT-PCR products were then cloned into a TOPO vector (Thermo Fisher, Waltham MA, USA).

For each parent, 150 bacterial transformants (grown on antibiotic-coated agar plates) were isolated for subsequent study, based on three independent rounds of low-cycle-number RT-PCR and cloning. By employing two-tailed unpaired Student’s t-test, we identified a trend in the father of higher expression for the wild type allele, although it did not reach significance (defined as *p* < 0.05) (Fig. 1e). However the results from the mother were significant (*p* < 0.03), and even more so when the data for both parents were combined (*p* < 0.01). This strongly suggested that RNA from the mutant allele was undergoing degradation due to NMD surveillance.

### Discussion and conclusions

ARL6IP1 is an integral transmembrane protein and its main function lies in ER shaping. It contains hairpin loop domains that localize to smooth ER tubules [5, 9]. ER-shaping proteins play an intrinsic role in the network of mitochondrial organization in motor neurons. Studies have shown the role of ARL6IP1 in elongation of axonal mitochondria, suggesting that loss of function variants in ER-shaping proteins disrupt mitochondrial network organization in motor neurons [7].

Homozygous variants in *ARL6IP1* were previously reported in patients with spastic paraplegia, diffuse sensory and motor polyneuropathy and acromutilation. These patients had late onset of disease and were diagnosed at 14 months, and by 10 years they could walk with support but with unsteadiness and scissors gait. They had skeletal deformities (loss of terminal digits) but cognition was normal [10]. In another study a patient was born to second-cousin parents with reduced head circumference (31.5 cm), and presented with acromutilation and congenital insensitivity to pain with small fibers involvement as additional features [8]. Crawling began at 14 months and the patient was able to walk with support at 4.5 years of age. The variant reported here has been recently identified in another family [11]. However, the phenotype found in our two patients is more severe than all other reported ARL6IP1 patients to date. It is intriguing to speculate on the reasons why. Clinical workup for both affected did not point to any perinatal infections. We cannot rule out confounding environmental factors or genetic modifiers. We note that there is a family history among relatives of skeletal dysplasia and early infantile death. A subset of variants from the WES data (filtered by zygosity and ACMG classification) are provided in Table 1. Some of these may act as genetic modifiers, for example *ARMC4* and *HYDIN* are both associated with autosomal recessive primary ciliary dyskinesia, and although the variants are heterozygous they may have played a role in the newborns’ respiratory distress. Similarly the *NCKAP1* and *RBMX* variants may have exerted an effect on neuronal function.

**Table 1:** List of variants potentially acting as genetic modifiers

GENE	Chr.	Start	End	Ref	Alt	Zygosity	ACMG	Variant	OMIM
NCKAP1	Chr2	183,847,656	183,847,656	C	A	Het	Pathogenic	Splice site	
HEY1	Chr8	80,678,495	80,678,501	GCATGTG	–	Het	Likely Pathogenic	Removes initiation codon of transcript NM_001282851.1	
PABPC1	Chr8	101,721,934	101,721,934	T	–	Het	Likely Pathogenic	PABPC1:uc003yjs.1:exon8:c.998delA:p.K333 fs	
ADCK5	Chr8	145,617,536	145,617,550	GGGGTGCAAGGTGAG	–	Het	Likely Pathogenic	ADCK5:uc003zch.3:exon12:c.1258_1267del:p.G420 fs	
ARMC4	Chr10	28,142,253	28,142,253	–	GCAT	Het	Likely Pathogenic	ARMC4:uc010qds.2:exon13:c.1635_1636insATGC:p.N546 fs	Ciliary dyskinesia, primary, 23 [autosomal recessive]
WNK1	Chr12	974,310	974,310	–	C	Het	Likely Pathogenic	WNK1:uc021qst.1:exon9:c.2174dupC:p.P725fs	Pseudohypoaldosteronism, type IIC [autosomal dominant]; Neuropathy, hereditary sensory and autonomic, type II [autosomal recessive]
GXYLT1	Chr12	42,499,814	42,499,817	GTAA	ATT	Het	Likely Pathogenic	GXYLT1:uc001rmt.4:exon4:c.574_577AAT	
HYDIN	Chr16	70,896,017	70,896,017	A	–	Het	Likely Pathogenic	HYDIN:uc031qwy.1:exon69:c.11711delT:p.I3904fs	Ciliary dyskinesia, primary, 5 [autosomal recessive]
RBMX	ChrX	135,960,147	135,960,147	–	AA	Het	Likely Pathogenic	RBMX:uc004fad.1:exon4:c.314_315insTT:p.P105fs	?Mental retardation, X-linked, syndromic 11, Shashi type [X-linked recessive]
MCF2	ChrX	138,699,671	138,699,671	C	A	Het	Pathogenic	Splice site	

Here we present a rare form of HSP characterized by cerebral abnormalities (failure to thrive, developmental delay, atrophy, leucoencephalopathy, generalized hypotonia, spasticity, partial agenesis of the corpus callosum), jejunal stricture, patent foramen ovale, patent ductus, distinct dysmorphic features, respiratory distress, culminating in respiratory insufficiency and neonatal death. Dysmorphic features included microcephaly, plagocephaly, overriding sutures, small fontanels, prominent nasal bridge, thin lips, retrognathia, high arched palate, long hands, long fingers, hyper-extendable joints, lumbosacral hyperlordosis, and large feet.

The presence of a rapidly progressive, complicated and fatal HSP broadens the clinical image associated with *ARL6IP1* variants, outlining the severe end of the phenotypic spectrum for mutations in this gene.

## Data Availability

Genotyping and whole exome sequencing data are available from the corresponding author on written request.

## Abbreviations

ER: Endoplasmic reticulum
GERD: Gastro-esophageal reflux disease
HSP: Hereditary spastic paraplegia
MAF: Minor allele frequency
RT: Reverse transcriptase
SPG: Spastic paraplegia gene
VCF: Variant caller file
